# Effectiveness of antidepressants: an evidence myth constructed from a thousand randomized trials?

**DOI:** 10.1186/1747-5341-3-14

**Published:** 2008-05-27

**Authors:** John PA Ioannidis

**Affiliations:** 1 Clinical Trials and Evidence-Based Medicine Unit, Department of Hygiene and Epidemiology, University of Ioannina School of Medicine and the Biomedical Research Institute, Foundation for Research and Technology-Hellas, Ioannina, Greece; 2 Institute for Clinical Research and Health Policy Studies, Department of Medicine, Tufts University School of Medicine, Boston, USA

## Abstract

Antidepressants, in particular newer agents, are among the most widely prescribed medications worldwide with annual sales of billions of dollars. The introduction of these agents in the market has passed through seemingly strict regulatory control. Over a thousand randomized trials have been conducted with antidepressants. Statistically significant benefits have been repeatedly demonstrated and the medical literature is flooded with several hundreds of "positive" trials (both pre-approval and post-approval). However, two recent meta-analyses question this picture. The first meta-analysis used data that were submitted to FDA for the approval of 12 antidepressant drugs. While only half of these trials had formally significant effectiveness, published reports almost ubiquitously claimed significant results. "Negative" trials were either left unpublished or were distorted to present "positive" results. The average benefit of these drugs based on the FDA data was of small magnitude, while the published literature suggested larger benefits. A second meta-analysis using also FDA-submitted data examined the relationship between treatment effect and baseline severity of depression. Drug-placebo differences increased with increasing baseline severity and the difference became large enough to be clinically important only in the very small minority of patient populations with severe major depression. In severe major depression, antidepressants did not become more effective, simply placebo lost effectiveness. These data suggest that antidepressants may be less effective than their wide marketing suggests. Short-term benefits are small and long-term balance of benefits and harms is understudied. I discuss how the use of many small randomized trials with clinically non-relevant outcomes, improper interpretation of statistical significance, manipulated study design, biased selection of study populations, short follow-up, and selective and distorted reporting of results has built and nourished a seemingly evidence-based myth on antidepressant effectiveness and how higher evidence standards, with very large long-term trials and careful prospective meta-analyses of individual-level data may reach closer to the truth and clinically useful evidence.

## Background

Few drugs have been as successful blockbusters as the class of antidepressants. Cumulatively, hundreds of millions of patients have taken these medications, and the selective serotonin reuptake inhibitors (SSRIs) and newer generation drugs in particular have been immensely popular. Antidepressants reflect one of the major manifestations of medicalization of modern society [1]. In 2006, 5 of the 35 drugs with top sales in the USA were antidepressants, and each of them had sales of 1.08–2.25 billion dollars in that year (Table 1) [2]. About 30% of the cost of depression in the USA (80 billion dollars per year) goes to drug expenditures [3].

**Table 1:** Top-selling antidepressants in the USA, 2006

**Drug (brand name)**	**Rank across all drugs**	**Sales (billions $)**
Venlafaxine XR (Effexor XR)	6	2.25
Escitalopram (Lexapro)	10	2.10
Sertraline (Zoloft)	15	1.77
Bupropion XL (Wellbutrin XL)	16	1.67
Duloxetine (Cymbalta)	35	1.08

Information is derived from [2]

This is not an epidemic that lacks evidence-based material to support it. Few drugs have had such a long chain of double-blind, placebo-controlled trials performed to demonstrate their effectiveness and to pass through seemingly strict regulatory approvals. The randomized literature of antidepressants is apparently one of the richest in evidence-based credentials. While for a large proportion of medical interventions, we have no or few clinical trials ever conducted, for antidepressants there are probably well over a thousand. PsiTri, an online library of clinical trials for mental health conditions [4], lists 4058 clinical trials for depression, and a large share of them (over a quarter of the total, exact count depends on eligibility criteria) pertain to randomized trials of antidepressants. A systematic review of SSRI trials for diverse indications until 2003 [5] found 702 trials (411 comparisons between SSRIs and placebo, 220 comparisons between SSRIs and tricyclic antidepressants, and 159 comparisons between SSRIs and active therapies other than placebos or tricyclic antidepressants). In another review [6] of 12 antidepressants where only double-blind, placebo-controlled trials for diverse indications in adults were involved, sponsors furnished data to the FDA on 406 trials with approximately 100,000 randomized patients.

Formally, statistically significant benefits have been repeatedly demonstrated and the medical literature is flooded with several hundreds of "positive" trials, both pre-approval and post-approval. In theory, this is a prototype of evidence-based medicine where treatments pass through rigorous randomized testing, and are vastly successful both in the clinical science arena as well as in the market.

This picture of bliss was questioned recently by two large, well-conducted meta-analyses [7,8]. In this review, I examine first what these meta-analyses have found and what are some possible limitations of these studies. Then I try to dissect what are the components that have constructed the seemingly evidence-based picture that antidepressants are so effective and why this picture may be problematic on close scrutiny. I try to address eventually whether antidepressants are widely indicated to treat depression and whether it is unethical to kill a living myth. Finally, I make some suggestions about how we can get appropriate evidence on these drugs.

### Upset by meta-analyses

#### Selective reporting

The first meta-analysis used data that were submitted to the U.S. Food and Drug Administration (FDA) for 12 antidepressant drugs that were approved between 1987 and 2004 [7]. These were bupropion SR (Wellbutrin SR, GlaxoSmithKline), citalopram (Celexa, Forest), duloxetine (Cymbalta, Eli Lilly), escitalopram (Lexapro, Forest), fluoxetine (Prozac, Eli Lilly), mirtazapine (Remeron, Organon), nefazodone (Serzone, Bristol-Meyers Squibb), paroxetine (Paxil CR, GlaxoSmithKline), sertraline (Zoloft, Pfizer), venlafaxine (Effexor, Wyeth), and venlafaxine XR (Effexor XR, Wyeth). The major advantage of using data submitted to FDA is that this includes all the trials that each company had registered as evidence in support for marketing approval or change in labelling. This registration allows one to have knowledge of all these trials, regardless of whether they were eventually published or not. Moreover, the process of regulatory review is such that there is less room for manipulating analyses and distorting results in data entered in the FDA registry tables.

The meta-analysts found 74 eligible FDA-registered trials with 12,564 patients. Among them, a third (n = 26 trials [31%] with 3449 patients) had remained unpublished. The FDA had determined that half of the registered trials (38/74) had found statistically significant benefits for the antidepressant ("positive" trials). All but one of these trials had been published in journals. Conversely, of the other half trials (36/74) that were deemed to be "negative" by the FDA, one in three were published as "negative" results; another 11 trials were published, but the results were presented in such a way so as to seem "positive" and 22 "negative" trials were silenced and never appeared in the literature.

The meta-analysts studied the estimated effectiveness of these drugs when data were combined from the FDA records and when data were combined from the published literature. For all drugs, the published literature inflated the effect sizes. The inflation varied from 11% to 69% and it was 32% on average. The FDA data would suggest that these agents had small, modest benefits (standardized effect size [ES] = 0.31 on average). Conversely, for 4 of the 12 agents, if one were to perform unawares only a meta-analysis of the published data, the summary result would suggest clinically important effectiveness (ES>0.5). This was not true for any agent based on more complete FDA data.

#### Treatment and placebo effect as a function of baseline severity

A second meta-analysis in *PLoS Medicine *used also data that were submitted to FDA on 6 new generation antidepressants and eventually used the information on four of them (5 trials on fluoxetine, 4 on venlafaxine, 8 on nefazodone, and 16 on paroxetine) [8]. For 2 other drugs (sertraline and citalopram), some trials were simply reported even in the FDA databases as having non-significant results. In contrast with the other meta-analysis, the investigators of this meta-analysis did not wish to impute data when there was such missing information.

The *PLoS Medicine *meta-analysis asked the question: is there a relationship between the baseline severity of depression and the difference in effectiveness between drug and placebo? Meta-regression analyses identified such a relationship. Drug-placebo differences were generally small, but they increased with increasing baseline severity. The meta-analysts used a previous consensus [9] to propose that a clinically important difference needs to be at least 3 points in the Hamilton scale or ES>0.50. The difference between drug and placebo became large enough to be clinically important only in the small minority of patient populations with severe depression (baseline score exceeding 28 in the study population). Even in these severely depressed patients, the difference between drug and placebo was due to the fact that placebo became less effective; there was no evidence that the antidepressants became more effective. The authors concluded that most of the benefit from antidepressants is duplicated by the placebo effect. This is a conclusion that had been proposed also based on earlier meta-analysis [9]. Moreover, the current meta-analysis added the insight that these agents may be of clinical use only in severely depressed people, a small minority compared with the vast populations who take antidepressants currently. Even in the few extremely depressed patients, the eventual benefit was due to lack of responsiveness of placebo, not due to increased responsiveness to antidepressants.

#### Limitations in the meta-analyses

**Figure 1:**
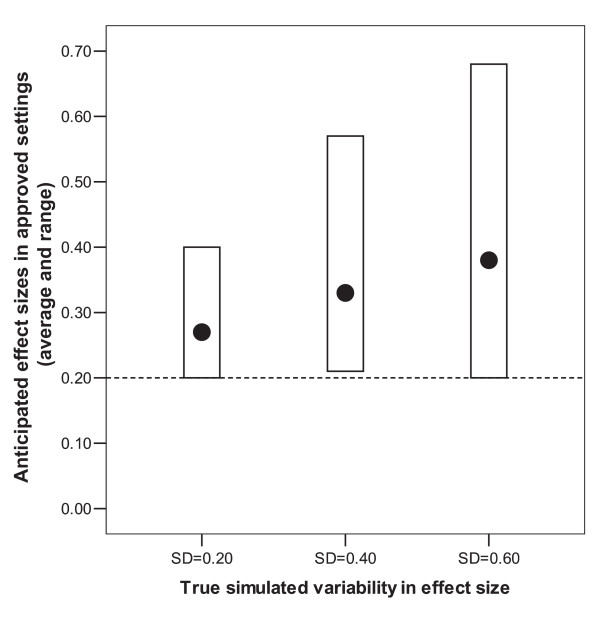
Anticipated mean and range of average effect size (ES) for trials in countries where a drug is approved, when the true average effectiveness is ES = 0.20 and the standard deviation (SD) of the estimated ES across trials is 0.20, 0.40, and 0.60. Trials are assumed to be of similar size and similar weight in the calculations. Forty approval packages with 5 trials each have been simulated in each of the three settings and the data show the ES in the successful packages (those where the average ES is at least 0.20).

Both meta-analyses have some limitations. Many more trials are conducted after approval or outside of the FDA approval process. Moreover, registries of approved agents do not include antidepressants that were possibly tested in clinical trials in the USA, but did not make it (presumably because of more "negative" results), although they made it and were approved in other countries, e.g. fluoxamine, milnacipran, or mianserin. Among antidepressive drugs tested in the USA, only the "luckier" ones, the ones with larger ES, went to the FDA and received approval. The lack of a comprehensive global database is a major deficit in that we may be missing trials done in countries where the overall results for a particular agent were not very promising or overtly negative. Figure 1 shows a simple simulation: suppose that a drug is tested in 40 countries and 5 small trials are preformed for licensing purposes in each country. Let us suppose that on average the drug has a true effect that is small (ES = 0.20). Each of the perfectly unbiased studies is expected to find on average ES = 0.20 and there can be some variability. We can examine situations with different levels of variability around this average, corresponding to standard deviations of 0.20, 0.40, and 0.60. The smaller the trials and the larger the diversity of the populations and drug response, the more variability is expected around the mean of ES = 0.20. Suppose the drug is approved only in countries where the 5 trials show average ES at least 0.20. This is expected to happen in about half the countries. Figure 1 shows what the average ES estimates are in the trials registered in countries where the drug was approved: ES is markedly inflated. Similar considerations apply, if we consider not only many countries, but also many drugs tested in many countries.

Even focusing on FDA-registered trials, their data are not necessarily totally unbiased. Inherent biases in the study design and analysis cannot be corrected by simple registration. Data collection, arbitration of measurements and outcomes and multiple analysis options leave room for selectivity and for presentation of more optimal conclusions – even in FDA-registered results. Second, even these data are eventually incomplete in important details. This is amply demonstrated by the considerable number of studies that were simply registered as having "negative" results without further details on effect sizes, and by the additional missing information that the meta-analysts had to impute even for FDA-registered data. Third, these trials did not have available individual-level information and the data collection and arbitration of outcomes and measurements remained out of reach of the meta-analysts. For the considerable proportion of patients who did not complete the trials, typically last observation carried forward (LOCF) methods were applied, but these have limitations and may lead to overestimation of treatment effects in some circumstances [10].

All these limitations are more likely to have resulted in inflation of the treatment benefit, although there is considerable uncertainty about the exact bias. Of note, the *PLoS Medicine *meta-analysis [8] noticed funnel plot asymmetry, i.e. smaller trials had larger effects than larger trials. Funnel plot asymmetry is typically considered a sign of publication bias (small "negative" trials remaining unpublished), but this is clearly a misleading simplification [11]. Here publication bias in theory is impossible for FDA-registered data. The authors attributed the asymmetry to confounding due to higher severity scores in smaller trials [8]. However, an alternative explanation is that even for FDA-registered trials, results may still be biased. Exclusion or inclusion of specific patients and data due to questionable eligibility criteria or grey measurements, selection of imputation techniques, use or not or adjustments, and selective reporting of outcomes allow for manipulation in effect size estimation. In small trials, the same amount of manipulation will inflate the effect size more than in large trials, in other words the *vibration *of the effect size is larger [12,13]. The FDA review process will of course decrease analytical flexibility, but evaluation of depression involves messy outcomes and analyses are not cut in stone. In all, if anything, expectation of these biases further reinforces the message about antidepressants being less effective than thought.

A more serious limitation is inherent in the use of meta-regression techniques in the *PLoS Medicine *meta-analysis. The main analysis used a fixed effects meta-regression, and only a secondary analysis used a mixed effects approach. The latter, which may be more appropriate than fixed effects [14], had less conclusive results. Meta-regression modelling can be biased [15]. When the trials have only small differences in the average values of severity (as in this case), the slope of the regression terms can be affected by outliers and leverage problems. The most important limitation stems from the ecological fallacy [16,17]. The regression used as a moderator variable the average baseline severity of depression in each group of participants in each trial arm. However, this is a proxy that does not represent equally well all participants. For example, the average baseline score may be 28, but this may include patients with scores of 17, 27, 32, and 36. The relationship may not have been the same, if data could have been analyzed for individual patients. This is to say, while net effectiveness (difference of drug from placebo) seemed to increase with increasing average severity, within a specific trial it could be that the effectiveness decreased with increasing severity. Ecological fallacy is the main reason why meta-regression analyses with group average are viewed with scepticism [17].

Finally, the *PLoS Medicine *meta-analysis describes the end of the severity spectrum in the analyzed trials as containing patients who are "most extremely depressed" or alternatively "very severe" depression. In fact, "very severe" depression would correspond to patients with even worse depression status, primarily those hospitalized because of major depression. In fact, the analyzed regulatory trials have typically avoided including hospitalized patients with truly so extreme depression, because these newer agents had been shown early on to be ineffective – or at least less effective than older agents – in such patients.

### Understanding the construction of the myth of antidepressant effectiveness

Acknowledging these caveats, the lessons we get from these meta-analyses and from the previous literature on antidepressants show us that there are many components that have helped to create the impression that antidepressants are very effective and worthy of being so popular in the general population.

#### Statistical versus clinical significance

The typical trial in the antidepressant field is a small investigation with anywhere between a few dozen and a few hundreds of participants. Nevertheless, these trials have used outcomes that can pick formally statistically significant differences between the compared arms even with such small sample sizes. The typical choice is depression scales such as the Hamilton Rating Scale of Depression. Such continuous outcomes can show formally statistically significant results (p < 0.05) even for differences that are small and trivial. Statistical significance is confused with clinical significance. A consensus by the National Institute for Clinical Excellence (NICE) has suggested that at least a 3 point difference is needed in the Hamilton scale or equivalently ES = 0.50 to claim a clinically important effect [9]. Nevertheless, the results of several single trials with statistically significant results and the results of practically all meta-analyses with statistically significant results *exclude *that and ES = 0.50 can be conferred by antidepressants, when we examine the 95% confidence intervals of the effects. Here one should acknowledge that there is nothing absolute about the cut-off of ES = 0.50 and some investigators may disagree with this cut-off. Typically proposed thresholds in the literature for small, moderate and large effects for continuous outcomes are 0.2, 0.5, and 0.8 standard deviations, but even this is arbitrary [18].

#### Study design and selection of study populations

The industry of randomized trials of antidepressants has generated over the years a long chain of practices and design "standards" that aims to maximize the chances of showing larger benefits from given drugs. These practices and standards include, but are not limited to, the use of placebo-controls, placebo lead-in periods, and a set of exclusion criteria for recruited participants.

Placebo-controls are of course dictated by regulatory agencies, and there is some considerable justification for them. Empirical data, even before the meta-analyses discussed above, have repeatedly shown that the placebo effect in depressive symptoms is large, variable, and seemingly increasing in magnitude in more recent trials [19]. However, this also generates the paradox that although we acknowledge, approve and massively sell drugs that we consider to be more effective than placebo, we continue to perform trials that require some of the participants to take what we consider to be ineffective treatments [20]. As a compromise, some trials randomize patients 2:1 or even 3:1, 4:1 and 5:1 to active drug versus placebo, a contradiction to the sense of equipoise that should permeate the ethics of running a clinical trial. Obviously, the placebo-controlled trials have a better chance of showing larger benefits in terms of absolute effects; head-to-head comparisons typically show no or very small differences in overall efficacy of one antidepressant over the other, regardless of whether old or new agents are involved [21-23]. For example, a meticulous systematic review of fluoxetine versus other antidepressants concluded than even if some nominally significant results were seen in some comparisons, "the clinical meaning of these differences is uncertain, and no definitive implications for clinical practice can be drawn" [21].

The use of placebo lead-in excludes patients who show a good response to placebo over a brief period of time, before participating in the proper trial. In theory, this may inflate the difference between drug and placebo in the patients who are eventually enrolled in the trial, if the good response to placebo in the lead-in period correlates with good response to placebo also during the longer follow-up of the trial. The presence and strength of this correlation is debatable [24].

Finally, the corpus of antidepressant trials has silently adopted a series of exclusion criteria. An empirical evaluation found that common exclusion criteria are short-episode duration, mild severity of illness, psychiatric comorbidities, long duration of illness, medical comorbidities, and prior non-response to treatment [24]. Actually, there is little empirical evidence that any of these criteria, perhaps with the exception of short-episode duration, does affect the magnitude of the net treatment effect. Conversely, they all diminish the generalizability of the trial findings. Based on their extensive sales, we have to infer that antidepressants are used far more widely in clinical practice compared with the narrow clinical trial setting defined by such restrictive eligibility criteria.

Overall, we know little about how design features can influence results. For example, even the dosing schedule (flexible versus fixed), the number of treatment arms, and the percentage of female patients have been reported to be associated with the magnitude of the treatment effect in trials [25].

#### Short follow-up

Most antidepressant trials have limited duration of follow-up, typically 6 weeks, and rarely exceeding 8 weeks. Some trials even last only 3–4 weeks. Even with such short follow-up, attrition (losses to follow-up, and/or discontinuation of study medication) is very common in these trials [26]. Imputing outcomes when information is missing due to attrition is not easy and leaves room for bias.

It is mostly differences in long-term, hard clinical outcomes that would matter (suicide, loss of job, other major personal or social events), because these drugs are sometimes used by patients in the community on a far more long-term basis. The evidence on long-term maintenance has been reviewed by a meta-analysis of trials where patients responding at the acute phase have been randomized to maintenance versus placebo [27]. While large reduction in the odds of relapse has been demonstrated, these 31 trials are still small (total n = 4410), most do not exceed 12 months of follow-up, they focus on those who responded acutely, and have not been able to address other hard outcomes of depression.

#### Selective and distorted reporting

As demonstrated by the *New England Journal of Medicine *meta-analysis [7], selective and distorted reporting of results is a major problem in the antidepressant literature. Even with all the manipulations listed above and with potentially selective analyses, only half of the trials of antidepressants reach conventional statistical significance. The other half either disappear or are further distorted so as to be published with the impression that they also have found "positive" results. Meta-analyses of the published literature are thus likely to give misleading impressions about the effectiveness of these drugs.

#### Unknown harms

Antidepressant trials are not geared towards demonstrating the possible harms of these medications. The imbalance of emphasis between effectiveness and harms in the design and reporting of randomized trials has been repeatedly demonstrated in various medical specialties [28], including mental health interventions [29]. Small trials are unlikely to pick any major harms, even relatively common ones, let alone uncommon harms that are life-threatening and may lead to death. Antidepressants are thus licensed in almost perfect vacuum on harms information. Harms may be detected subsequently from meta-analyses, large registries, or other means of post-marketing surveillance, but all of these methods have limitations and imperfect sensitivity and specificity. The large debate about the unrecognized suicide risk for children taking antidepressants is one example about how very important harms can go unrecognized [30-32]. The late addition of black box warnings increases the sense of uncertainty about these drugs [33]. Uncertainty about harms does not help anyone, even the industry. The reputation of the industry can be ruined even by postulated harms that may not exist, e.g. as in the debate about increase in suicides in adults.

#### Extension of the market

As described above, clinical trials of antidepressants enrol highly restricted types of patient populations, and typically employ short-term administration. However, once licensed, the antidepressant drugs are used widely in the general population and are often prescribed for very long-term use [1-3]. Antidepressants are a prime example of the over-medicalization of our society [1]. Direct to consumer advertisement probably contributes extensively to this diffusion of use. A pharmacoeconomic evaluation found that direct to consumer advertisement of antidepressants may result in treating people so widely that 94% of antidepressant use due to direct-to-consumer advertising is from non-depressed individuals [34]. The same evaluation concluded that this is more than counterbalanced by the accumulated societal benefits conferred by the treatment of truly depressed patients who would not have been treated otherwise [34]. However, this inference is based on an assumption of large effectiveness of antidepressants that is probably not commensurate with the current evidence.

#### Building the supporting scientific myth

Besides randomized trials, the industry has used biological arguments to promote the idea in the wider community that antidepressants have mechanisms of action that correct major chemical imbalances in the brain [1]. The typical example is SSRI marketing based on the serotonin hypothesis. It is unfair or even wrong to summarize decades of neuroscience research as showing that depression is caused by the imbalance of one or another neurotransmitter (serotonin, norepinephrine, or other) in the brain. If anything, evolving knowledge of biology shows that depression is an extremely complex behavioural phenotype regulated by a large number of biological pathways, external exposures, and genetic factors, each one contributing a small effect [1,35]. Drugs that supposedly impact on a single pathway are expected to have small impact on the overall biology of depression and would be equilibrated by balancing changes. Clinical effects would thus also be expected to be small on average.

### Answering the main practical questions

#### Are antidepressants indicated in depression?

Based on the above considerations, antidepressants are probably indicated only in select patients with major depression, probably preferentially in those who have severe symptoms and have not responded to anything else. For most patients with some depressive symptoms who are currently taking antidepressants, using these drugs would not have been the preferred option, placebo would be practically as good, if not better, and would save toxicities and cost.

Current approval of specific antidepressants by regulatory agencies means that some specific criteria have been met that demonstrate some nominally statistically significant results in some trials and for some scales, but this is not equivalent to proof of major clinical benefit, effectiveness in the wider population level. The trials that are undertaken are not necessarily scientific exercises but rather exercises that are designed to produce a demonstration of a particular effect for regulatory/legal purposes.

Some other meta-analysts have found even more gloomy results than the two meta-analyses reviewed above. For example, after including both published and unpublished data (29 and 11 trials, respectively), one meta-analysis recently found absolutely no benefit from paroxetine over placebo when focusing on the "hard" clinical outcome of treatment discontinuation [36]. Some treatment effect probably exists in "softer" scale-based outcomes, but it is likely to be small, and possibly the effect estimates are inflated compared to the truth. Cumulative meta-analyses suggest that for drug treatments in mental health indications in general, treatment effects tend to decrease over time, as more studies are conducted [37]. Empirical evidence suggests also that much of the placebo effect in antidepressant trials is related simply to the number of patient visits during a trial [38]. Trials where patients are scheduled to visit physicians more often for evaluation during the study improve more, regardless of whether they are given drug or placebo. There is a 0.6–0.9 point improvement in the Hamilton scale for each additional visit performed during the trial.

Perhaps most people given antidepressants for depressive symptoms would just need some attention from their physician and people to talk to and take some care of them. Antidepressants may be covering largely the lost placebo of human interaction and patient-physician interaction that has become so sparse in modern society. However, I will not comment here whether formal psychotherapies are as or more effective than antidepressants. The evidence base of psychotherapies is not any better, it pertains to similarly small trials, affected by most of the same problems (and more), and it would need a whole separate review on its own [39].

#### Is it unethical to kill a living myth?

If most of the antidepressant efficacy reflects simply the placebo effect, and if most people just benefit as much as the placebo effect allows, is it unethical to kill a living myth? One might argue that if the general population is informed that antidepressants are not really effective, this might demolish the benefits that we get from the placebo effect when we administer these drugs. However, is it not unethical to lie to patients that an intervention is effective when it is not? Moreover, if we want to utilize this placebo effect, why is it justified that this should cost cumulatively more billion dollars to society than almost any other (truly effective) pharmaceutical intervention for any other condition? It would be weird for our society to compromise with a view that someone should make fortunes by selling official placebos.

The pharmaceutical industry is going through rough times [40]. As the exclusive licenses of most major blockbusters are expiring currently, the fear is that unless the big pharma manages to keep up in its profits, then its R&D effort will be stalled. An even more cynical view is that since our society aims at making profit, if we don't allow the pharmaceutical industry to make large profits, even by selling largely ineffective drugs to the population at-large, then some other industry will attract capital investments and will flourish instead. This may be in an entrepreneurial domain that is less useful or even very harmful for society, e.g. the weapons industry. My reply is that the pharmaceutical industry should be encouraged and supported to conduct high-quality research in antidepressant drugs and beyond. Serious investigation in the life sciences by corporate, government and academic investigators should be given priority as societal goals, but expectations should be realistic: most major promises do not materialize [41], major discoveries are rare, drugs are not miraculous, and we should be honest about how much has been achieved and how much we can achieve. Telling lies to compete against more unscrupulous producers of lies in our society is not the way to make scientific progress.

## Conclusion

After the publication of the *PLoS Medicine *meta-analysis, Eli Lilly, the producers of fluoxetine made a statement pointing attention to the fact that "more than 40 million people suffering from depression have been treated with fluoxetine in over 100 countries" [42]. Fluoxetine received approval from FDA based on 5 registered trials where a total of 817 patients with depression took fluoxetine [4]. A simple calculation shows that for each randomized patient receiving fluoxetine in the FDA application package, approximately 50,000 patients have been treated subsequently in everyday practice. Even if we consider all the other trials of fluoxetine (pre- and post-approval and with various indications), the ratio is likely to remain way above 1,000 to 1. Similar considerations apply practically to all antidepressants. While we have a large number of trials, they are all small. Typically these trials do not provide sufficient evidence to determine the exact benefit with any accuracy in specific types of patients, e.g. as a function of the baseline risk of depression [43]. Moreover, they say little about hard clinical outcomes and the long-term benefit-harm ratio.

No matter how careful the efforts to collate small trials, the interpretation of the available fragmented evidence will remain largely subjective. For example, the authors of the *New England Journal of Medicine *meta-analysis wrote an editorial in the BMJ where they said that compared to the more negative stance of the *PLoS Medicine *meta-analysis, their stance was more critical rather than dismissive [44]. To settle this important matter with robust evidence, we need large trials with 100-fold more patients than what has been the norm to-date. These trials should be linked to long-term follow-up registries with thorough recording of long-term outcomes for both efficacy and harms. Efficacy outcomes should include long-term management and evolution of depression, suicide and major life events. These trials should be designed in a way that they do not simply have power to show a nominally statistically significant benefit (of debatable clinical importance), but for demonstrating whether the benefit is conclusively within the range of clinical importance and has a long-term portend. These trials should also have sufficient power to answer this question not only in the overall study population, but also in specific risk strata and as a function of the baseline risk of severity. When hundreds of millions have been treated with these drugs with such a suboptimal evidence base, asking to conduct mega-trials of 50,000 participants for each antidepressant agent is not an exaggerated wish. If there are concerns that different agents or different classes thereof may have different effects, then large-scale evidence should be collected for each of them and for head-to-head comparisons thereof. The general public should be sensitized not to compromise with suboptimal evidence for drugs that are used by hundreds of millions of people.

Antidepressant trials should be pre-registered before their conduct [45], but this alone does not suffice. Their protocols need to be entirely transparent and their analysis plans explicit in detail upfront. There should be no room for flexibility in the collected data and performed analyses. The full data should be available in public, for scrutiny and use by all interested scientists. Finally, the complete agenda of these trials should be co-ordinated in a way that they will eventually be part of an overarching global prospective meta-analysis of individual-level data. Simply waiting to perform meta-analyses after the fact with data that are selectively analyzed, presented, submitted, and published is an invitation to confusion. Under such circumstances, meta-analyses end up being mostly detective tools that struggle to unearth problems under dark circumstances. Conversely, full registration of large-scale transparent trials with full detailed availability of data and prospective meta-analyses would show that we are serious about finding the true merits and risk-benefit of antidepressants or any other type of medical intervention.

Clinical trial registration has made major progress [45], but details of protocols are still missing and flexibility and selective analyses and reporting are probably still prevalent. This does not mean that this vision is utopian. In fact, it may be the only way to avoid running into vicious circles of pseudo-evidence-based medicine where we are blinded by a blizzard of small, selectively designed, analyzed and reported trials. This may sound depressing for the current state of clinical trials research on antidepressants and beyond. Nevertheless, even if one feels a bit depressed by this state of affairs, there is no reason to take antidepressants, they probably won't work.

## Competing interests

The author declares that he has no competing interests.

## Authors' contributions

JI conceived this article, carried out the included analyses, and wrote the paper.

**About the author: **John PA Ioannidis, MD, PhD, born in New York, NY in 1965, is Professor and Chairman of the Department of Hygiene and Epidemiology, University of Ioannina School of Medicine and has an adjunct appointment as Professor of Medicine at Tufts University School of Medicine. He is interested in research methodology, evidence-based medicine, clinical and molecular epidemiology, and mathematical modelling. He has published approximately 400 peer-reviewed publications and is a member of the editorial board of 18 peer-reviewed journals.
